# Serum 25-Hydroxyvitamin D Level Is Positively Associated with Vascular Reactivity Index in Patients with Type 2 Diabetes Mellitus

**DOI:** 10.3390/nu16111575

**Published:** 2024-05-23

**Authors:** Bang-Gee Hsu, Yi-Cheng Wang, Du-An Wu, Ming-Chun Chen

**Affiliations:** 1 School of Medicine, Tzu Chi University, Hualien 97004, Taiwan; gee.lily@msa.hinet.net (B.-G.H.);; 2 Division of Nephrology, Buddhist Tzu Chi General Hospital, Hualien 97004, Taiwan; 3 Division of Metabolism and Endocrinology, Buddhist Tzu Chi General Hospital, Hualien 97004, Taiwan; 4 Department of Pediatrics, Buddhist Tzu Chi General Hospital, Hualien 97004, Taiwan

**Keywords:** 25-hydroxyvitamin D, endothelial dysfunction, type 2 diabetes mellitus, vascular reactivity index

## Abstract

Circulating 25-hydroxyvitamin D (25(OH)D) significantly influences endothelial function. This study assessed the correlation between serum 25(OH)D and endothelial function using the vascular reactivity index (VRI) in patients with type 2 diabetes mellitus (T2DM). Fasting blood samples from 102 T2DM participants and VRI were assessed. Patients were divided into three categories based on VRI: low (VRI < 1.0), intermediate (1.0 ≤ VRI < 2.0), and good (VRI ≥ 2.0). Among these patients, 30 (29.4%) had poor, 39 (38.2%) had intermediate, and 33 (32.4%) exhibited good vascular reactivity. Higher serum fasting glucose (*p* = 0.019), glycated hemoglobin (*p* = 0.009), and urinary albumin-to-creatinine ratio (*p* = 0.006) were associated, while lower prevalence of hypertension (*p* = 0.029), lower systolic blood pressure (*p* = 0.027), lower diastolic blood pressure (*p* < 0.001), and lower circulation 25(OH)D levels (*p* < 0.001) were associated with poor vascular reactivity. Significant independent associations between diastolic blood pressure (*p* = 0.002) and serum 25(OH)D level (*p* < 0.001) and VRI were seen in T2DM patients according to multivariable forward stepwise linear regression analysis. Serum 25(OH)D positively correlated with VRI values, and lower levels of serum 25(OH)D were linked to endothelial dysfunction in T2DM patients.

## 1. Introduction

Type 2 diabetes mellitus (T2DM) is a chronic metabolic disorder recognized as a vascular and metabolic disease that significantly impacts world health [1]. By 2045, there will likely be 693 million adults worldwide with T2DM, up from 451 million in 2017 [2]. The progression of diabetes eventually involves cardiovascular disease (CVD), causing major deaths in patients with T2DM worldwide [3].

Endothelial dysfunction (ED) is a non-traditional cardiovascular (CV) risk factor associated with inflammatory mechanisms that cause CV events in T2DM. ED is the earliest vascular defect in the early stages of atherosclerosis that develops in the DM population [1]. Previous studies have identified ED as an independent predictor of poor prognosis in DM patients with either microvascular or macrovascular complications [1,4]. ED-related impaired endothelium-dependent vasodilation, decreased bioavailability of nitric oxide (NO), and prothrombotic tendencies are strongly associated with CVDs [1,5].

Understanding ED mechanisms in T2DM is crucial, as factors in DM affecting ED are likely multifaceted. Many recent investigations have demonstrated the role of 25-hydroxyvitamin D (25(OH)D) in vascular pathology, both in vitro and in vivo [6,7]. Hypovitaminosis D is associated with increased ED and activation [8]. Vitamin D insufficiency is associated with CV disease risk and ED in individuals diagnosed with T2DM [9]. Studies indicate that vitamin D possesses antioxidant properties and is essential for modulating proinflammatory cytokines in the vascular endothelium, decreasing the expression of endothelial adhesion molecules, and potentially aiding in endothelium repair [7]. However, the DIMENSION trial revealed that a tailored 16-week regimen of vitamin D supplementation led to minor but not statistically significant improvement in endothelial function in a T2DM cohort [10]. In an effort to elucidate the influence of serum total 25(OH)D levels on ED and its relationship with the same in T2DM patients, we conducted a study to explore the association between circulating levels of 25(OH)D and ED, quantified by the vascular reactivity index (VRI), in individuals diagnosed with T2DM.

## 2. Materials and Methods

### 2.1. Study Design and Participants

From January to March 2019, 102 patients with T2DM diagnosis for more than 6 months at an eastern Taiwan Medical Center were enrolled. The Hualien Tzu Chi Hospital, Buddhist Tzu Chi Medical Foundation’s Research Ethics Committee granted ethical permission (IRB106-108-A). All the participants gave their informed consent in writing. Ages > 18 years and a diagnosis of diabetes mellitus (DM) based on fasting serum glucose levels ≥126 mg/dL, a 2 h oral glucose tolerance test ≥200 mg/dL, or the use of oral hypoglycemics or insulin were the inclusion criteria. Exclusion criteria included limb amputation, heart failure, acute coronary syndrome, acute myocardial infarction, malignancy, acute infection, or refusal of consent.

### 2.2. Anthropometric and Blood Pressure Measurements

Body weights and heights of the participants were recorded with a precision of 0.5 kg and 0.5 cm, respectively. Waist circumference was determined by measuring the mid-point between the lower ribs and the hip bones, with the participants placing their hands on their hips. The body mass index (BMI) was computed by dividing the weight in kilograms by the square of the height in meters [11,12]. Blood pressure was measured in the morning after a 10 min seated rest using standard mercury sphygmomanometers. Systolic blood pressure (SBP) and diastolic blood pressure (DBP) were recorded thrice at 5 min intervals and averaged for analysis. Hypertension was ≥140/90 mmHg or recent antihypertensive medication use in the preceding two weeks [11,12].

### 2.3. Biochemical Investigations

Post an 8 h overnight fast, around 5 mL of venous blood was drawn from the patients and promptly centrifuged at 3000× *g* for a duration of 10 min. Serum levels of blood urea nitrogen (BUN), creatinine, fasting glucose, glycated hemoglobin (HbA1c), total cholesterol, triglycerides, and low-density lipoprotein cholesterol (LDL-C) were assessed using a Siemens Advia 1800 autoanalyzer (Siemens Healthcare GmbH, Henkestr, Germany) [11,12]. A random spot urine test assessed the urinary albumin-to-creatinine ratio (UACR). Commercial enzyme-linked immunosorbent assays determined the human serum total 25(OH)D levels (Crystal Chem USA, Elk Grove Village, IL, USA) [13]. The Chronic Kidney Disease Epidemiology Collaboration formula was utilized to compute the estimated glomerular filtration rate (eGFR).

### 2.4. Endothelial Function Measurements

An FDA-approved digital thermal monitoring (DTM) equipment (VENDYS-II; Endothelix, Inc., Houston, TX, USA) was used to measure endothelial function parameters following an overnight fast and abstinence from tobacco, alcohol, caffeine, and vasoactive medicines. After a 30 min supine rest in a room with a temperature range from 22 °C to 24 °C, the right upper arm was subjected to blood pressure cuffs, and the left index finger was used as a control, while the right finger was used for occlusion. For the DTM, 5 min of cuff stabilization, 5 min of cuff inflation, and 5 min of cuff deflation were included for both hands. Reactive hyperemia was induced in the fingertip by quickly inflating the right upper arm cuff to 50 mmHg above the systolic pressure for five minutes. VRI, computed using VENDYS software (https://www.vendys2.com/, accessed on 14 May 2024), ranges from 0.0 to 3.5, indicating the reactive hyperemia’s maximum temperature differential between the rebound and zero reactivity curves. VRI < 1.0 indicated poor reactivity, from 1.0 to 1.9 indicated intermediate reactivity, and ≥2.0 indicated good reactivity [11].

### 2.5. Statistical Analysis

IBM SPSS (version 19.0; IBM Corp., Armonk, NY, USA) was used for all analyses. The Kolmogorov–Smirnov test was used to assess variable distributions. Normally distributed data were compared using Student’s independent t-tests (two-tailed), presented as means ± standard deviations. The Mann–Whitney U test was used to analyze non-normally distributed data, presented as medians and interquartile ranges. The Kruskal–Wallis analysis was used to compare groups (poor, intermediate, and good VRI) for non-normally distributed parameters (fasting glucose, triglyceride, HbA1C, BUN, creatinine, and UACR). In contrast, one-way ANOVA was used for normally distributed data. Univariate and multivariate logistic regression analyses assessed the total 25(OH)D levels for vascular reactivity dysfunction (intermediate/poor) or poor vascular reactivity. The receiver operating characteristic (ROC) curve analysis was used to determine the total 25(OH)D levels indicative of vascular reactivity dysfunction or poor vascular reactivity. Log transformation ensured the normality of fasting glucose, triglyceride, HbA1C, BUN, creatinine, and UACR levels. The chi-square test was used to compare categorical variables. After variables correlated with VRI were found using simple linear regression, relevant variables were included to a multivariable forward stepwise regression analysis. A *p*-value of <0.05 was deemed statistically significant.

## 3. Results

Table 1 outlines the clinical characteristics and use of antihypertensive and antidiabetic medication in the complete T2DM cohort, comprising 102 patients. Among them, 30 (29.4%) patients exhibited poor VRI, 39 (38.2%) exhibited intermediate VRI, and 33 (32.4%) exhibited good VRI. Significant differences between the groups revealed a reduced prevalence of hypertension (*p* = 0.029), lower SBP (*p* = 0.027) and DBP (*p* < 0.001), elevated serum fasting glucose (*p* = 0.019), HbA1C (*p* = 0.009), UACR (*p* = 0.006), and lower serum total 25(OH)D levels (*p* < 0.001) in those with poorer VRI. No notable variations existed among the groups in age, sex, or the utilization of antihypertensive, antilipid, or antidiabetic medications.

Table 2 illustrates the multiple logistic regression analysis of serum total 25(OH)D levels in patients with DM, assessing vascular reactivity dysfunction or poor reactivity. Each 1 ng/mL rise in serum total 25(OH)D corresponded to a 15% (*p* = 0.004) and 12% (*p* = 0.016) risk reduction, respectively.

The ROC curve for serum total 25(OH)D levels predicting vascular reactivity dysfunction had an AUC of 0.762 (95% CI = 0.659–0.866, *p* < 0.001), while for poor vascular reactivity, the AUC was 0.674 (95% CI = 0.562–0.786, *p* = 0.0023) (Table 3). According to the Youden index, the optimal cutoff total 25(OH)D level for predicting vascular reactivity dysfunction was 14.94 ng/mL (sensitivity: 69.57%; specificity: 75.76%; positive predictive value: 85.72%; and negative predictive value: 54.35%), and the optimal cutoff total 25(OH)D level for predicting poor vascular reactivity was 13.38 ng/mL (sensitivity: 66.67%; specificity: 63.89%; positive predictive value: 43.48%; and negative predictive value: 82.15%), respectively (Table 3).

**Figure 1:**
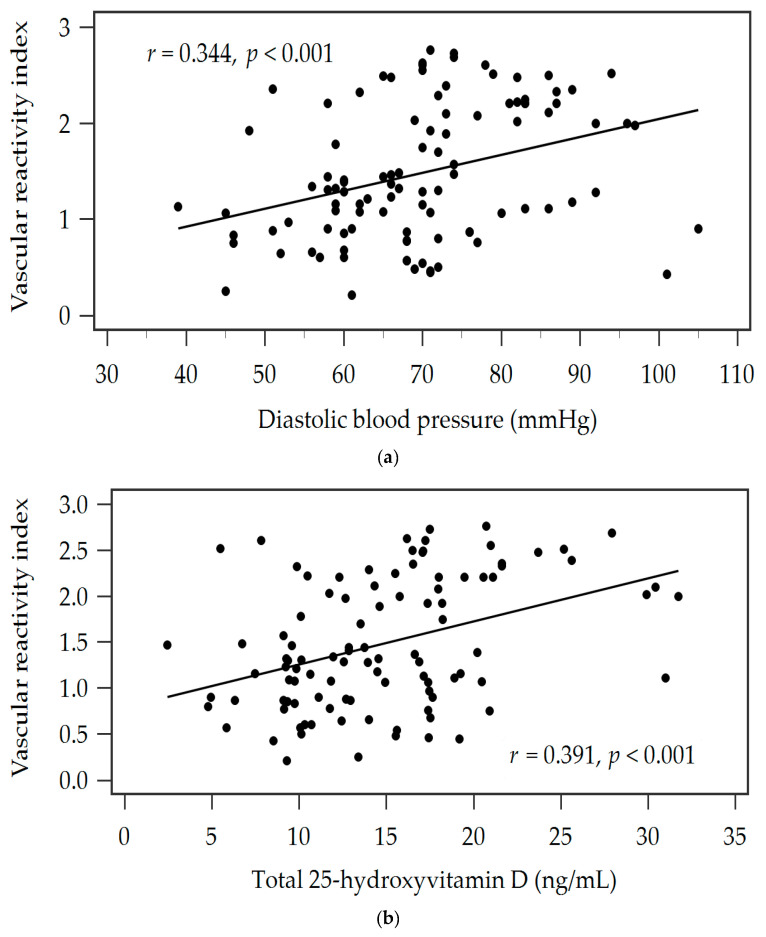
Associations between vascular reactive index (VRI) and (**a**) the diastolic blood pressure (mmHg), or (**b**) the levels of 25-hydroxyvitamin D (ng/mL) in 102 patients with type 2 diabetic mellitus.

Table 4 shows the VRIs and clinical features of 102 T2DM patients. A negative connection was seen between T2DM VRI and serum log-transformed glucose (log-glucose, *r* = −0.199, *p* = 0.045), log-HbA1C levels (*r* = −0.249, *p* = 0.012), and log-UACR (*r* = −0.207, *p* = 0.037). The total 25(OH)D levels (*r* = 0.392, *p* < 0.001), SBP (*r* = 0.214, *p* = 0.031), DBP (*r* = 0.344, *p* < 0.001), and hypertension (*r* = 0.202, *p* = 0.042) all showed positive correlations. DBP (β = 0.282, adjusted R^2^ change = 0.070, *p* = 0.002) and serum total 25(OH)D levels (β = 0.342, adjusted R^2^ change = 0.146, *p* < 0.001) were found to be independent predictors of VRI in patients with T2DM. Figure 1a,b displays the two-dimensional scattered plots of VRI values in relation to DBP and serum total 25(OH)D levels in patients with T2DM.

## 4. Discussion

Our study revealed positive associations between serum 25(OH)D levels, hypertension, SBP, DBP, and VRI in T2DM patients. Conversely, serum fasting glucose, HbA1C levels, and UACR showed negative associations. In T2DM patients, VRI showed significant and independent relationships with serum total 25(OH)D levels and DBP, even after controlling for confounders in patients with T2DM.

Validated endothelial function measures are markers of CV diseases and major cardiac events [14]. ED has been proposed as a core pathway linking diverse risk factors to vascular complications [15]. It has become popular to use noninvasive tools or biomarkers to evaluate ED, the early atherosclerotic event, in order to predict unfavorable outcomes in T2DM patients [16]. DTM of vascular reactivity is a noninvasive technique that measures the temperature response of the skin as a surrogate for blood flow, providing an indirect assessment of endothelial function. During DTM, a temperature sensor detects changes in skin temperature following a brief period of ischemia. The VRIs, derived from DTM data, quantify the change in temperature and, thus, the blood flow response. A higher VRI indicates better endothelial function, which is crucial for maintaining vascular health and is inversely related to cardiovascular risk factors and disease. Recently, DTM-derived VRI has emerged as a simple, operator-independent, and noninvasive approach to evaluate microvascular function in patients [17].

Evidence from multiple sources supports the significant link between hypertension, hyperglycemia, renal dysfunction, and ED [5,18,19]. ED is closely associated with hypertension, and numerous studies have supported the effectiveness of antihypertensive therapy in improving ED [20]. Within the Framingham Offspring Cohort, a direct correlation was observed between hypertension intensity and endothelial function deterioration [20]. In contrast, this study highlights elevated SBP and DBP in patients with T2DM and good vascular reactivity compared with those with intermediate or poor reactivity. Vascular dysfunction due to autonomic neuropathy is frequent in diabetes. Impaired sympathetic and parasympathetic vasomotor control contributes to diabetic autonomic neuropathy, prevalent in approximately 7.4% of patients with T2DM, and increases with age [6,21]. Previous research suggested that in glaucoma patients, vascular dysregulation may lower SBP and DBP [6]. In our simple regression analysis, SBP and DBP revealed a positive correlation with VRI. Notably, after multivariate regression analysis among patients with T2DM, DBP remained significantly associated with VRI. This may suggest poor ED is associated with autonomic neuropathy.

Evidence indicates that vascular ED begins early in arteriosclerosis and is observable during early glucose intolerance in patients with T2DM [5]. Additionally, DM contributes to ED progression [5,11], and a study also reported that some classic hypoglycemic agents may ameliorate both ED and hyperglycemia [22]. Earlier studies indicate that chronic hyperglycemia induces ED through varied pathways: heightened reactive oxygen species (ROS) from hyperglycemia, elevated transforming growth factor-β and plasminogen activator inhibitor-1, and diminished NO availability impacting both macrovascular and microvascular function [1] As expected, our study found elevated fasting glucose and HbA1C level are inversely related to vascular reactivity in T2DM patients.

Increased CV risk has been associated with albuminuria, an early ED sign and an important factor in diabetic kidney disease, in a number of clinical studies, including T2DM patients [23]. Biologically, UACR suggests an association between systemic microcirculation damage and is implicated in ED in diverse disorders such as dementia and neuropathy [24,25]. In this study, patients with T2DM with impaired vascular reactivity exhibited a notably elevated UACR, implying a correlation between declining renal and endothelial function.

In cardiometabolic medicine, hypovitaminosis D is associated with DM, elevated blood pressure, heart failure, myocardial infarction, and stroke [14]. Serum 25(OH)D is also associated with inflammatory responses, oxidative stress, and endothelial function [26]. Vitamin D deficiency is linked to vascular ED, as observed in both in vitro and in vivo studies [27]. In vitro studies showed that vitamin D can impede the process of endothelium-dependent vasodilation and affect vascular smooth muscle cell proliferation by reducing vasodilator availability [28]. Animal studies showed that vitamin D receptor-deficient knockout mice exhibit reduced NO production, which resulted in abnormal vascular function, including raised pulse and aortic augmentation pressures. Analysis of the aorta tissue showed elevated amounts of collagen and lower levels of elastin, indicating arterial stiffening [29]. Hypovitaminosis D may elevate the levels of intact parathyroid hormone, leading to arterial hypertension, unfavorable vascular remodeling, and calcification of the vascular wall [13]. Epidemiological research has revealed a reverse relationship between levels of 25(OH)D and the velocity of the carotid-femoral pulse wave in the general populace, senior individuals, and patients recently diagnosed with hypertension [13]. This relationship also holds for the thickness of the aortic intima-media in cases of subclinical atherosclerosis [30], as well as the heightened arterial stiffness and vascular calcification observed in patients suffering from chronic kidney disease [13].

T2DM patients often exhibit reduced serum 25(OH)D levels [27]. Severe vitamin D deficiency doubles the risk of mortality from all causes and CV diseases in patients with T2DM [31]. T2DM patients deficient in vitamin D exhibit limited brachial flow-mediated dilation, a helpful measure of vascular endothelial function [32]. A recent study by Tanaka et al. highlighted a noteworthy link between the reactive hyperemia index and circulating 25(OH)D levels, suggesting ED related to vitamin D deficiency can manifest in the early stages of T2DM. They established a connection between circulating 25(OH)D concentrations and endothelial functionality, pinpointing <16.5 ng/mL as an indicator of ED in patients with poorly controlled T2DM [27]. Our findings revealed a positive correlation between serum 25(OH)D levels and VRI values in T2DM patients. For intermediate and poor vascular reactivity, serum 25(OH)D levels were 13.56 ± 5.00 ng/mL and 12.17 ± 4.34 ng/mL, respectively, consistent with the mentioned study. In addition, childhood vitamin D insufficiency correlates with adult ED [33].

There are certain constraints to our research. First, our cross-sectional observational study, conducted at one hospital, does not allow for the determination of the causal relationship between serum 25(OH)D concentrations and ED in patients with T2DM. Factors such as diverse diets, supplement use, physical activity, and the timing of vitamin D sampling might introduce bias [13,34]. Prospective studies on a large scale and over an extended period are required to explore the causality. Second, while serum 25(OH)D is a standard indicator of vitamin D status, its accuracy depends on vitamin D binding protein (VDBP) [35]. Approximately 90% of circulating vitamin D binds to VDBP, affecting its usability in cellular functions [36]. Recent findings link VDBP gene polymorphisms to varied VDBP levels, potentially explaining racial disparities in total 25(OH)D concentrations despite similar free vitamin D levels [34]. Third, numerous studies have demonstrated significant disparities in plasma 25(OH)D concentrations between genders. However, in our research, we found that the serum total 25(OH)D concentrations in T2DM patients did not significantly differ between males and females (mean total 25(OH)D concentrations: 14.68 ± 5.56 ng/mL for males and 14.96 ± 6.69 ng/mL for females, *p* = 0.827). Estrogen exerts considerable influence on vitamin D metabolism, particularly among women of reproductive age. Additionally, women typically exhibit higher total fat mass than men, potentially resulting in the sequestration of fat-soluble vitamin D in adipose tissue and consequently reducing the bioavailability of vitamin D3 [37]. These gender-related differences necessitate consideration in future studies investigating VRI and endothelial function. Fourth, most participants in our study were over 65 years old, and only seven patients in this study had a serum of 25(OH)D level > 20 ng/mL, limiting the generalizability to younger and sufficient 25(OH)D serum concentrations in T2DM populations. Further studies or randomized controlled trials with larger sample sizes, gender-related differences, or more sufficient serum 25(OH)D concentrations are needed to better clarify the causal relationship between 25(OH)D levels and ED in T2DM patients.

## 5. Conclusions

In patients with T2DM, this study observed a direct link between serum 25(OH)D concentrations and ED, as assessed using the VRI. Additionally, the VRI displayed significant independent correlations with circulating 25(OH)D levels and DBP. Future prospective studies are imperative to ascertain the causal relationship between serum 25(OH)D levels and ED in patients with T2DM.

## Figures and Tables

**Table 1:** Clinical features of diabetes mellitus patients as determined by the vascular reactivity index using digital thermal monitoring.

Characteristics	All Patients (*n* = 102)	Good Vascular Reactivity (*n* = 33)	Intermediate Vascular Reactivity (*n* = 39)	Poor Vascular Reactivity (*n* = 30)	*p* Value
Age (years)	64.61 ± 9.27	63.09 ± 8.94	66.49 ± 8.08	63.83 ± 10.86	0.262
Height (cm)	161.38 ± 9.21	159.43 ± 9.42	162.23 ± 9.60	162.43 ± 8.36	0.336
Body weight (kg)	71.25 ± 14.15	72.63 ± 14.92	69.08 ± 10.99	72.54 ± 16.83	0.482
Body mass index (kg/m^2^)	27.21 ± 4.16	28.47 ± 4.64	26.09 ± 2.70	27.29 ± 4.82	0.051
Vascular reactivity index	1.48 ± 0.70	2.34 ± 0.22	1.37 ± 0.27	0.68 ± 0.20	<0.001 *
Systolic BP (mmHg)	133.83 ± 17.91	137.61 ± 15.49	136.23 ± 18.37	126.57 ± 18.21	0.027 *
Diastolic BP (mmHg)	69.84 ± 12.95	77.12 ± 10.36	67.00 ± 11.95	65.53 ± 13.72	<0.001 *
Total cholesterol (mg/dL)	156.44 ± 34.64	153.70 ± 37.35	149.67 ± 27.35	168.27 ± 37.99	0.073
Triglyceride (mg/dL)	123.00 (81.00–170.25)	129.00 (86.00–208.50)	136.00 (81.00–170.00)	105.50 (71.00–163.50)	0.346
LDL-C (mg/dL)	85.47 ± 29.21	82.85 ± 28.93	81.41 ± 27.47	93.63 ± 31.00	0.187
Fasting glucose (mg/dL)	138.50 (112.75–170.25)	131.00 (110.00–141.50)	147.00 (118.00–180.00)	156.50 (120.25–183.25)	0.019 *
Glycated hemoglobin (%)	7.35 (6.48–9.15)	7.00 (6.20–7.50)	7.70 (6.60–9.10)	7.90 (6.58–10.13)	0.009 *
Blood urea nitrogen (mg/dL)	17.00 (13.00–23.00)	15.00 (13.00–21.00)	19.00 (14.00–24.50)	17.00 (13.00–23.00)	0.197
Creatinine (mg/dL)	1.10 (0.90–1.40)	1.00 (0.90–1.45)	1.10 (1.00–1.30)	1.30 (0.90-1.83)	0.290
eGFR (mL/min)	65.44 ± 22.71	71.26 ± 26.51	63.22 ± 16.92	61.94 ± 24.23	0.197
UACR (mg/g)	182.21 (81.873–311.77)	121.27 (28.61–270.81)	167.40 (63.30–315.48)	231.28 (157.65–390.50)	0.006 *
Total 25(OH)D (ng/mL)	14.76 ± 5.90	18.54 ± 6.33	13.56 ± 5.00	12.17 ± 4.34	<0.001 *
Female, n (%)	31 (30.4)	10 (30.3)	12 (30.8)	9 (30.0)	0.998
Hypertension, *n* (%)	58 (56.9)	22 (66.7)	25 (64.1)	11 (36.7)	0.029 *
ARB use, *n* (%)	53 (52.0)	19 (57.6)	22 (56.4)	12 (40.0)	0.294
β-blocker use, *n* (%)	18 (17.6)	6 (18.2)	7 (17.9)	5 (16.7)	0.986
CCB use, *n* (%)	33 (32.4)	10 (30.3)	14 (35.9)	9 (30.0)	0.834
Statin use, *n* (%)	51 (50.0)	16 (48.5)	19 (48.7)	16 (53.3)	0.910
Fibrate use, *n* (%)	32 (31.4)	11 (33.3)	13 (33.3)	8 (26.7)	0.804
Metformin use, *n* (%)	64 (62.7)	22 (66.7)	23 (59.0)	19 (63.3)	0.795
Sulfonylureas use, *n* (%)	54 (52.9)	17 (51.5)	24 (61.5)	13 (43.3)	0.317
DDP-4 inhibitors use, *n* (%)	59 (57.8)	21 (63.6)	22 (56.4)	16 (53.3)	0.692
Thiazolidinedione use, *n* (%)	10 (9.8)	4 (12.1)	4 (10.3)	2 (6.7)	0.762
SGLT2 inhibitors use, *n* (%)	23 (22.5)	7 (21.2)	9 (23.1)	7 (23.3)	0.975
Insulin use, *n* (%)	31 (30.4)	9 (27.3)	11 (28.2)	11 (36.7)	0.671

Continuous variables are presented as the mean ± standard deviation and were examined using one-way variance. Non-normally distributed variables are expressed as the median and interquartile range and were evaluated using the Kruskal–Wallis test. Categorical values are expressed as a number (percentage) and were analyzed using the chi-square test. 25(OH)D, 25-hydroxyvitamin D; ARB, angiotensin receptor blocker; BP, blood pressure; CCB, calcium channel blocker; DDP-4, dipeptidyl peptidase 4; eGFR, estimated glomerular filtration rate; LDL-C, low-density lipoprotein cholesterol; SGLT2, sodium glucose cotransporter 2; UACR, urine albumin-to-creatinine ratio. * *p* < 0.05 was considered statistically significant after the Kruskal–Wallis or one-way variance analysis.

**Table 2:** Multivariate logistic regression analysis of serum total 25-hydroxyvitamin D for vascular reactivity dysfunction (intermediate and poor vascular reactivity) or poor vascular reactivity in 102 diabetes mellitus patients.

Model	25(OH)D (per 1 ng/mL of Increase) for Vascular Reactivity Dysfunction		25(OH)D (per 1 ng/mL of Increase) for Poor Vascular Reactivity	
	OR (95% CI)	*p* Value	OR (95% CI)	*p* Value
Crude model	0.826 (0.751–0.909)	<0.001 *	0.878 (0.800–0.963)	0.006 *
Adjusted model	0.845 (0.753–0.948)	0.004 *	0.878 (0.790–0.976)	0.016 *

Adjusted model: adjusted for age, body mass index, diastolic blood pressure, estimated glomerular filtration rate, fasting glucose, glycated hemoglobin, hypertension, sex, systolic blood pressure, total cholesterol, urine albumin-to-creatinine ratio, and 25-hydroxyvitamin D. 25(OH)D, 25-hydroxyvitamin D; CI, confidence interval; OR, odds ratio. * *p* < 0.05 was considered statistically significant.

**Table 3:** 25-hydroxyvitamin D has a diagnostic role in vascular reactivity dysfunction (intermediate and poor vascular reactivity) or poor vascular reactivity.

	**Vascular Reactivity** **Dysfunction**						
	**AUC (95% CI)**	***p* Value**	**Cut-off**	**Sen (%)**	**Spe (%)**	**PPV (%)**	**NPV (%)**
25(OH)D (ng/mL)	0.762 (0.659–0.866)	<0.001 *	14.94	69.57	75.76	85.72	54.35
	**Poor Vascular Reactivity**						
	**AUC (95% CI)**	***p* Value**	**Cut-off**	**Sen (%)**	**Spe (%)**	**PPV (%)**	**NPV (%)**
25(OH)D (ng/mL)	0.674 (0.562–0.786)	0.0023 *	13.38	66.67	63.89	43.48	82.15

25(OH)D, 25-hydroxyvitamin D; AUC, area under the curve; 95% CI, 95% confidence interval; Sen, sensitivity; Spe, specificity; PPV, positive predictive value; NPV, negative predictive value. * *p* < 0.05 was considered statistically significant.

**Table 4:** Correlation between clinical factors and levels of the vascular reactivity index using univariable or multivariable linear analysis.

Variables	Vascular Reactivity Index				
	Simple Regression		Multivariable Regression		
	*r*	*p* Value	Beta	Adjusted r^2^ Change	*p* Value
Female	0.022	0.823	–	–	–
Hypertension	0.202	0.042 *	–	–	–
Age (years)	−0.063	0.527	–	–	–
Height (cm)	−0.172	0.084	–	–	–
Body weight (kg)	−0.004	0.970	–	–	–
Body mass index (kg/m^2^)	0.146	0.144	–	–	–
Systolic blood pressure (mmHg)	0.214	0.031 *	–	–	–
Diastolic blood pressure (mmHg)	0.344	<0.001 *	0.282	0.070	0.002 *
Total cholesterol (mg/dL)	−0.116	0.246	–	–	–
Log-Triglyceride (mg/dL)	0.086	0.392	–	–	–
LDL-C (mg/dL)	−0.171	0.085	–	–	–
Log-Glucose (mg/dL)	−0.199	0.045 *	–	–	–
Log-Glycated hemoglobin (%)	−0.249	0.012 *	–	–	–
Log-BUN (mg/dL)	−0.018	0.857	–	–	–
Log-Creatinine (mg/dL)	−0.142	0.156	–	–	–
eGFR (mL/min)	0.161	0.107	–	–	–
Log-UACR (mg/g)	−0.207	0.037 *	–	–	–
Total 25-hydroxyvitamin D (ng/mL)	0.392	<0.001 *	0.342	0.146	<0.001 *

Data pertaining to BUN, creatinine, fasting glucose, glycated hemoglobin, triglycerides, and UACR exhibited a skewed distribution and were subjected to log-transformation prior to analysis. The data were analyzed using either simple linear regression or multivariate stepwise linear regression, with the factors considered being hypertension, diastolic blood pressure, log-glucose, log-glycated hemoglobin, log-UACR, and total 25-hydroxyvitamin D. BUN, blood urea nitrogen; eGFR, estimated glomerular filtration rate; LDL-C, low-density lipoprotein cholesterol; UACR, urine albumin-to-creatinine ratio. * *p* < 0.05 was considered statistically significant.

## Data Availability

The data generated and analyzed in this study are all included in this publication.
